# Supplementary material for: Are carbon emissions trading and green financial instruments synergistic? -Comprehensive quantitative research based on content analysis
Source: PLoS One. 2024 Mar 7;19(3):e0298601. doi: 10.1371/journal.pone.0298601 (PMC10919601; doi:10.1371/journal.pone.0298601)
Supplement: S1 Data — (ZIP) [file pone.0298601.s001.zip › Data/地级市Z_F工作报告.zip!/─┌├╔╣┼/═¿┴╔╩╨/└┤╫╘╣1⁄2╓┌║┼í╛┬φ┐╦╩2╛▌═°í┐_2014.docx]

各位代表：

现在，我代表通辽市人民政府向大会报告工作，请予审议，并请市政协委员和列席会议的同志们提出意见。

一、过去一年工作的回顾

2013年是本届政府的开局之年，也是应对严峻考验并取得新成效的一年。我们深入贯彻党的十八大、十八届二中、三中全会和习近平总书记系列讲话精神，全面落实自治区“8337”发展思路和市委各项决策部署，统筹推进稳增长、调结构、促改革、惠民生，较好完成了四届一次人代会确定的各项工作任务，在推进开放转型、创业富民的道路上迈出了新步伐。

(一)经济发展稳中向好。

有效落实2013年24项重点工作行动计划，强化政策和项目支撑，实现了经济稳定增长。地区生产总值完成1811.8亿元，增长9.7%;地方财政总收入149.3亿元，增长8.1%。公共财政预算收入103.6亿元，增长16.2%;固定资产投资1577.7亿元，增长23.1%。农牧业基础地位稳步提升。粮食产量再创新高，达到132.6亿斤，总量全区第一。牧业年度牲畜存栏1888万头只，肉类产量稳居全区前列。全力稳定企业生产经营。落实电价补贴、税费减免、金融支持等综合措施，企业运行态势趋稳，工业经济实现有质量增长。全部工业增加值完成1036.2亿元，增长11.8%，其中规模以上工业增加值增长12.2%。服务业发展扩量提速。全市社会消费品零售总额完成372.4亿元，增长11%。外贸进出口总额实现翻番。文化旅游、商贸物流、金融等现代服务业加快发展。

(二)结构调整取得新进展。

围绕“五大基地”建设推动转型发展，产业内部结构进一步优化，三次产业联动发展能力增强。支柱产业集群化发展，铝新材料产业新增76个品种，原铝转化率达到80%以上。玉米生物科技产业新增64个品种，玉米精深加工转化率达到54%。镍铬合金项目形成10万吨产能，奠定了不锈钢全产业链发展的基础。能源结构调整实现新突破，新增风电并网15万千瓦，累计并网343万千瓦。光伏装机规模达到7万千瓦，并网运行6万千瓦。清洁能源发电量65亿度，增长34%，占发电总量的22%。大农业内部结构调整成效突出，以节水农业为标志，新增高产高效粮食功能区175万亩，节水农业集中连片规模位居全国首位，实现节水3.1亿立方米，亩均增收370元以上;以优质肉牛为重点，专业化、规模化养殖业加快发展。肉牛存栏达到254万头，新增26万头。大力发展种养结合，推进农牧业资源循环利用，秸秆转化利用率达73%。“三品一标”农产品认证面积新增80万亩，达到560万亩。在北京成功举办农产品博览会，科尔沁绿色安全食品品牌影响力不断扩大。科技创新能力明显增强，组织实施新型煤电铝、现代煤化工等6项科技攻关，创新发展能力不断提升。

(三)城乡统筹发展进程加快。

实施了一批产城融合、提质扩容项目，城镇综合承载能力和城市品位进一步提升。房地产业健康发展，各类在建工程达到1219万平方米。100个新农村新牧区示范村和70个幸福社区建设进展顺利。全市新增城镇人口3.8万人，城镇化率达到44.3%。加快发展县域经济和非公有制经济，旗县特色产业园区面积达到200平方公里，入驻企业突破1000户。新增非公有制经济市场主体3.8万户，增长34.6%。

(四)可持续发展能力不断增强。

列入自治区重点调度的30个项目，全部开工建设，完成投资465.7亿元。交通、水利、电力等基础设施建设成效明显。好通高速和三条一级路建成通车，市与旗县间实现高等级公路连接。新建农村牧区水泥路、沥青路1800公里，通畅率达到54.6%。新增7条国内航线，民航旅客吞吐量57.3万人次，增长67%。莫力庙水库分洪渠和分洪闸扩建完成基础工程。投资4.4亿元改造农村电网2428公里，开鲁500千伏输变电工程开工建设。实施百万亩城郊生态林、科尔沁沙地治理等重点生态工程，完成林业生态建设256万亩。主城区绿化力度加大，创建国家园林城市工作通过国家专家组初验。扎鲁特旗北部9个自然村生态移民搬迁顺利实施。加强环境监测和重点流域环境治理，大力发展循环经济，节能减排指标完成自治区下达任务。

(五)民生和社会事业推进力度加大。

城乡居民收入稳步增长，城镇居民人均可支配收入达到21009元，增长11.6%，增速全区第一;农牧民人均纯收入达到9621元，增长13.2%，均高于经济增长速度。农牧民生活质量明显提高，每百户农牧民家庭小汽车拥有量由5年前的4.4辆提高到9.8辆。全力扶贫攻坚，整合各类资金25亿元用于扶贫，实现1.8万户、7万农牧民稳定脱贫。实施创业就业工程，新增城镇就业1.8万人，高校毕业生就业或落实就业去向1.1万人，其中蒙语授课和贫困家庭高校毕业生就业比例达到25.8%，农村牧区劳动力转移就业49万人。推进百姓安居工程，全市建设各类保障性安居住房3.5万套，竣工1.9万套，保障房覆盖面达到25.5%，居全区前列。改造主城区老旧小区30个，改造农村牧区危房3.15万户。提高社会保障水平，企业退休人员养老金达到1656元，提高106元。城乡居民最低生活保障标准均提高10%以上。受灾群众、贫困大学生和特殊困难群体得到及时有效救助。投入1.89亿元向低收入农牧户每户发放1吨取暖用煤。全面发展社会事业，各级各类教育协调发展，教育教学质量有新提高。213所农村牧区幼儿园全部建成开园。与北京西城区开展了教育交流合作。新建18所苏木卫生院，旗县医院整体水平基本达到二甲医院标准。新农合实现市级统筹，引入商业保险，大病报销额度最高提高到20万元。北京医管局组织9家知名医院对我市医疗机构开展了帮扶活动。完善公共文化服务体系，实施文化惠民工程，广播电视户户通工程完成9万户。成功举办第10届中国蒙古族服装服饰艺术节暨蒙古族服装服饰大赛、哲里木赛马节，提升了科尔沁文化旅游品牌知名度。群众性体育活动蓬勃开展，举办全市第四届运动会，在全区第八届少数民族运动会上取得第二名的好成绩。人口和计划生育工作不断加强，妇女儿童、外事侨务、宗教事务、气象、地震、慈善、老龄等工作扎实推进。维护社会和谐稳定，有效推进普法和依法治理，全面推广嘎查村“532”工作法和社区网格化管理，积极排查化解社会矛盾纠纷。加强社会治安防控体系建设，有效打击各类治安和刑事犯罪，当年命案全部侦破。强化安全生产和食品药品监管，及时处置应急突发事件，科左后旗“4·22”抗震救灾和灾后重建取得成效。民族团结进步事业得到巩固和加强。支持民兵预备役、国防和军队现代化建设，军政军民实现融合发展。

(六)政府自身建设得到加强。

严格执行“八项规定”、“约法三章”等中央、自治区和市委相关规定，开展整风肃纪专项行动，清理超标公务用车和办公用房。市本级“三公”经费支出同比减少16.7%。市级230项行政审批事项减少168项，缩减率达到73%。加强政府部门日常监督管理，实行绩效考评，促进了工作落实。认真执行人大及其常委会决定，自觉接受政协民主监督，广泛听取各民主党派、工商联和无党派人士意见，认真办理人大代表议案、建议和政协委员提案。加强政府法制工作，强化行政领域建章立制，行政执法水平不断提高。扩大政务公开范围，加强行政监察、经济责任审计和社会监督。全市上下以转变作风的实际成效汇聚各方力量，营造了良好发展氛围。

各位代表，成绩来之不易，这是自治区党委、政府和市委正确领导的结果，是全市各族干部群众不懈奋斗的结果。我代表市人民政府，向全市各族干部群众，向所有关心支持通辽建设与发展的社会各界朋友，表示衷心的感谢!

在看到成绩的同时，我们也清醒地认识到经济社会发展中存在的问题。一是经济下行压力依然存在。受市场需求不足影响，主要工业品价格波动较大，经济增速放缓，一些优势产业利润空间收窄，财政增收压力加大。二是经济结构不够合理。传统产业、低端产业、资源型产业多，新兴产业、高端产业、科技资金密集型产业少。服务业依然是经济发展的短板，现代服务业处于起步阶段。县域经济和非公有制经济规模小、竞争力不强，城乡区域发展不平衡。三是基础设施建设相对滞后。区域性综合交通体系不够完善，能源电力输出通道建设相对滞后，水利设施建设与经济社会发展要求不相适应。四是民生改善和社会事业发展力度不强。城乡居民收入总体水平不高，增收门路和渠道不宽。教育、医疗、社会保障等民生工作投入力度还需要进一步加大，民生工作制度还需要进一步规范。五是落实工作的质量和效率需要进一步提高。个别政府部门和机关干部存在作风不实、效率不高的问题等等。对此，我们将采取有力措施，认真加以解决。

二、2014年的主要工作任务

2014年是贯彻落实党的十八届三中全会精神、全面深化改革的开局之年，是贯彻落实习近平总书记视察我区重要讲话精神，深入推进自治区“8337”发展思路的关键一年。国际国内发展环境仍然复杂而不确定，机遇与挑战并存，全市各族干部群众守望相助，按照市委提出的“三个如何”，坚定信心，迎难而上，确保通辽经济社会健康持续发展、赢在转型期。

今年政府工作的总体要求是：高举中国特色社会主义伟大旗帜，以邓小平理论、“三个代表”重要思想、科学发展观为指导，全面贯彻落实党的十八大、十八届二中、三中全会和习近平总书记系列讲话精神，认真落实自治区“8337”发展思路及市委四届六次、四届七次全委会暨全市经济工作会议各项部署，坚持稳中求进、改革创新、民生优先，推进重点领域和关键环节改革，推进结构调整和转型升级，推进新型工业化、新型城镇化和农牧业现代化，着力保障和改善民生，着力创新社会治理，着力加强生态文明建设，加快转变政府职能，切实提高经济发展质量和效益，不断增创我市转型发展、科学发展、率先发展的新优势。主要预期目标是：地区生产总值增长10%，其中工业增加值增长12%;固定资产投资增长15%;公共财政预算收入增长8%;社会消费品零售总额增长12%;城镇居民人均可支配收入和农牧民人均纯收入分别增长10%和11%。

今年，重点抓好以下十个方面的工作：

(一)深化重点领域和关键环节改革，增强发展内生动力

根据自治区党委贯彻落实《中共中央关于全面深化改革若干重大问题的决定》意见和市委提出的“十大”改革任务，加快推进各项改革。一是充分激发市场主体活力，推动产业转型升级。重点推进工商登记改革，对在我市注册登记的各类市场主体，实行统一的登记程序和登记要求，降低市场准入门槛。推进霍煤集团等国有企业完善现代企业制度，鼓励非公有制经济参与国有企业改制重组，发展混合所有制经济，增强国有企业活力。完善国有资产管理体制改革，实行经营性国有资产集中统一监管，提高国有资产收益水平。着力转变经济发展方式，促进产业延伸升级。二是构建开放型经济新体制，打造内陆沿边开放、产业集群发展新高地。重点打造三大产业集群新高地，强化三条大通道建设，构筑两大开放平台，加快融入国内国际两个市场，培育开放竞争的新活力、新优势。三是深化农村牧区改革和扶贫攻坚，构建新型农牧业经营体系。进一步推进多种形式的适度规模经营，发展专业大户、家庭农牧场、专业化合作社和农牧业企业，鼓励和引导工商资本到农村牧区发展适合企业化经营的现代种养业。组建农牧业投资公司，提高农牧业资金使用效率。创新扶贫工作方式，实施精准化扶贫。四是推进具有区域特色新型城镇化，健全城乡发展一体化体制机制。创新主城区建设管理体制机制，推进综合执法。抓好户籍制度改革，放开城关镇和建制镇落户限制，有序放开主城区落户限制，加快农牧民转移人口市民化。五是深化行政管理体制改革，建设法治政府、服务型政府。进一步简政放权，旗县市全部建成政务服务中心和公共资源交易中心，提高基层服务站点覆盖面。加大政府购买服务力度，凡属事务性管理服务，引入竞争机制，通过合同、委托等方式向社会购买。六是强化权力运行制约，推进廉政机制创新和健全改进作风常态化制度。推行政府及其部门权力清单制度，依法公开权力运行流程，接受社会监督。七是创新文化体制机制，增强地区文化软实力。开展社会主义核心价值观教育活动，整合科尔沁文化旅游资源，推动文化与旅游融合发展。鼓励非公有制企业参与文化旅游产业发展，增强发展活力。八是推进社会事业改革和人才工作创新，着力保障和改善民生。重点推进城乡义务教育资源均衡配置，促进教育公平。实施卫生和人口计划生育机构改革，实施一方是独生子女的夫妇可以生育两个孩子的政策。九是创新社会治理体制，确保社会和谐稳定。加快构建城乡社区服务网络，全面推进幸福社区建设。培育和优先发展服务类社会组织。实行网上受理信访制度，涉法涉诉信访纳入法治轨道解决。十是加强生态文明制度建设，增强可持续发展能力。实行资源有偿使用制度和生态补偿制度，提高资源利用效率，推进霍林河国家级循环经济示范园区建设，实现绿色、低碳、循环发展。

(二)扩大有效需求，促进经济稳定增长

着力加大投资。准确把握国家政策走向，积极争取中央、自治区预算内资金。发挥政府投资的杠杆作用，推进资源、资产、资本一体化运作，创新投融资体制机制，充分挖掘民间投资潜力。支持引导金融机构扩大融资规模，引进金融机构和组建准金融机构20家以上，促进财务公司、电子结算等金融结算平台落户通辽。继续发挥“中小微企业池”作用，加强银企对接，拓宽企业融资渠道。推进露天煤业20亿元定向增发和蒙药公司创业板上市工作。全年新增社会融资270亿元，总量突破1400亿元。加大对外开放和招商引资力度，促进产业和市场深度融合。新签约亿元以上项目100个，当年开工率达30%以上，全年招商到位资金700亿元以上。

着力推进重点项目建设。强化项目对稳增长的支撑作用，列入今年自治区重点调度的30个项目，实行“一对一”全程跟踪服务，确保项目投资到位。开工建设350个工业重点项目，完成固定资产投资850亿元以上。协调推进“引绰济辽”工程前期，开工建设毛都水库。积极协调霍林河至珠恩嘎达布其口岸铁路、通辽至京沈客专联络线项目申报工作;协调通辽至长春高速公路加快建设进度、辽宁境内平安地至阜新段道路提高等级，完成奈曼至营口高速公路联络线项目申报工作;开工建设通辽至鲁北高速公路、霍林河至阿力得尔一级路、省道304线舍伯吐至大兴二级路;建设完成省道101线巴彦胡硕至霍林河段二级公路;调整优化国内航线，启动口岸机场业务工作，积极谋划国际航线。霍林河支线机场建成投入使用;建设完成昌图至通辽天然气管道输送项目。盘活资产存量，对停产、半停产企业和已形成固定资产规模的项目，量身制定扶持措施，促使企业尽快复工建成投产。对没有市场需求和难以盘活的企业，积极引导其尽快转产或资产变现。全面落实减轻企业负担的各项政策，助力企业达产达效，形成新的增长点。

着力扩大消费和市场需求。认真落实国家各项惠民政策，增加城乡居民转移性收入、劳动者工资收入和家庭财产收入，有效提高消费能力。推进城乡消费流通体系建设，加快现代连锁商业发展，大力扶持电子商务，实施800家日用品农家店信息化改造，扩大农村牧区现代流通服务网络覆盖面。积极培育养老健康、家政服务等消费热点，不断拓展消费空间。规范整顿市场秩序，严厉打击商业欺诈、制假售假等行为，保护经营者和消费者权益，营造良好消费环境。实施向俄蒙开放战略，积极拓展国际市场，继续抓好肉牛、红干椒等外贸示范基地和梅花生物等外贸龙头企业，外贸进出口总额增长10%以上。

(三)着力转变经济发展方式，推动结构优化升级

加快发展新型工业化。发挥我市资源和市场竞争优势，抓住国家治理大气污染、化解产能过剩的机遇，对我市资源型产业进行转化升级，做好“转”和“升”的文章，构建多元发展、多极支撑的新型工业化体系。“转”就是加大新技术应用和技术改造力度，发展新能源、新材料等战略性新兴产业，加快构建符合国家产业政策、具有区域优势和市场竞争力的产业发展格局。福耀集团淘汰平板玻璃生产线，转建日产1000吨高档汽车玻璃生产项目，实现玻璃制造业整体转型。“升”就是延长资源型产业链，提高产品附加值。加快建设锦联铝板带箔、创源高强高韧铝合金等25个铝新材料项目，新增铝产品80种，铝精深加工产能达到160万吨;加快建设梅花三期、开鲁中圆玉米生物等6个重点项目，新增玉米深加工产品100种;加快发展现代蒙药产业，尽快形成1500吨蒙药生产能力，建成全国最大现代蒙药产业园。通过转型升级，加快传统产业新型化发展步伐。

推进“五大基地”建设。清洁能源输出基地，加快建设开鲁至科尔沁、科尔沁至新民500千伏风电输送通道，年内清洁能源发电量突破70亿度，构建火电、风力、光伏和生物质发电多点布局、多元消纳的能源开发利用格局，打造新能源示范城市。现代煤化工生产示范基地，推进煤制乙二醇、乙醇酸、煤制气等重点项目建设，打造产值超百亿元的现代煤化工产业。有色金属生产加工和现代装备制造等新型产业基地，抓住国家实施电解铝阶梯电价的机遇，发挥优势推进有色金属产业延伸升级，向高端产业发展。承接产业转移，发展非资源产业，提升风电设备、汽车模具等装备制造业水平。绿色农畜产品生产加工输出基地，推进主城区和各旗县市食品加工产业园、科尔沁牛业产业园等重点项目建设，扩大有机绿色无公害农畜产品生产加工能力，提高市场份额，打造安全食品科尔沁品牌。体现草原文化、独具北疆特色的旅游观光、休闲度假基地，以创建科尔沁文化旅游名城为契机，规划实施500公里科尔沁文化旅游景观大道，发挥文化旅游资源的集成效应与整合优势，提升科尔沁文化旅游魅力。全年接待游客600万人次，旅游收入增长25%以上。

积极发展现代服务业。把现代服务业作为调整产业结构的着力点，集中建设一批特色突出、带动力强的服务业集聚区。突出发展现代物流业，加快建设科尔沁综合物流、空港物流、开发区综合物流和木里图工业物流园区，推进各旗县市特色物流园建设，健全功能、做大规模，形成各具特色的物流产业集聚区;加快建设通辽内陆港和保税物流中心，扩大货运规模，实现海铁联运，发展“陆港经济”;加快建设万达广场、万力商业综合体等项目，形成布局优化的城市综合商圈;加快建设“云端智慧通辽”和开发区“云计算”产业园等项目，促进信息产业与新经济模式融合发展。制定我市电子商务发展规划，推进电子商务平台建设，不断增强各类消费产业的拉动能力。

提升非公有制经济发展水平。落实扶持政策，完善调度制度，及时掌握企业诉求、解决存在问题。加大金融支持力度，对现有融资担保公司进行有效整合，增强信用担保能力，设立小微企业贷款风险补偿金，满足中小企业担保需求。优化发展环境，实行统一的市场准入制度，制定非公有制企业进入特许经营领域具体办法，充分释放非公有制经济发展活力和潜能，实现市场主体总量增长25%以上。

(四)强化“三农三牧”工作，加快发展现代农牧业

增强粮食综合生产能力。更加重视以建设旱涝保收基本田和优质玉米基地为重点的粮食生产。落实最严格的耕地保护制度，强化基本农田保护。新建节水高产高效粮食功能区120万亩，累计达到600万亩。集成各类科技要素，提高单产，确保粮食总产稳步提升。推进“粮安工程”，加强粮食仓储设施建设和粮食收储工作，为保障国家粮食安全做贡献。

加快打造现代畜牧业。更加重视以农牧民增收为核心，加快传统畜牧业向现代畜牧业转变。大力发展农区畜牧业，提高畜牧业产值占大农业、农区畜牧业产值占畜牧业的比重。发展种养结合，建设优质牧草基地，种植青贮面积300万亩。加快优质肉牛示范区建设，年内肉牛存栏达272万头。完善畜牧业市场流通体系，重点抓好优质肉牛等10个专业牲畜交易市场和10个专业合作社。培育壮大科尔沁牛业等21个产业化龙头企业，加快推进畜产品加工转化增值，让农牧民更多分享产业利润，实现农牧民持续增收。

提高科技对农牧业增产增效的贡献率。更加重视以科技支撑、培育新型农牧民为重点的农牧业科技创新。组织实施优质玉米、优质肉牛、优质红干椒等重大农牧业科技攻关，加大农牧业先进实用技术推广应用。培育新型农牧民，狠抓嘎查村科技服务员和农牧民科技培训。启动新一轮农牧业种子工程，加快建立一批优势种子生产基地及品种示范基地。健全动物防疫责任体系，重点抓好“布病”等人畜共患疫病防控。加强农村牧区气象服务和气象灾害防御体系建设，提高农牧业防灾减灾能力。

全力抓好农畜产品质量安全。更加重视以标准化生产和产品监管为重点的食品安全。以满足吃得安全吃得好为导向，大力发展安全农畜产品。抓好“菜篮子工程”，新增设施农业面积8万亩。加快推进现代农牧业标准体系建设，建立肉牛、玉米、红干椒、荞麦等首批7个产业标准体系，建设首批10个农畜产品标准化示范基地，抓好30个重点农畜产品品牌，扩大农畜产品地理标志保护范围。加强农畜产品安全监测，实施全程质量可追溯，切断污染物进入农牧业生产的链条，落实从田间牧场到餐桌全过程监管责任，确保农畜产品质量安全，把我市建设成在全国叫得响的绿色农畜产品生产加工输出基地。

加快构建新型农牧业经营体系。坚持家庭经营在农牧业中的基础性地位，推进家庭经营、集体经营、合作经营、企业经营等共同发展的农牧业经营方式创新。允许农牧民以承包经营权入股发展农牧业产业化经营，支持承包经营权在公开市场上向专业大户、家庭农牧场、农牧民合作社、农牧业企业流转，发展多种形式规模经营。重点抓好科左中旗现代农业综合改革发展示范区和科尔沁区、开鲁县土地流转试点，为全市农村牧区改革积累新经验。

加强新农村新牧区建设。加快推进危房改造、安全饮水、嘎查村街巷硬化、村村通电、村村通广播电视、校舍建设、标准化卫生室、文化活动室、便民连锁超市、农村牧区常驻人口社会保障“十个全覆盖”为重点的新农村新牧区建设，努力让农牧民过上好日子。推广嘎查村“532”工作法，从源头上预防和减少社会矛盾，促进农村牧区和谐稳定。开展人居环境综合整治，营造文明整洁的村屯环境，努力建设美丽乡村。

(五)推进新型城镇化，促进城乡区域协调发展

优化城镇布局和形态。强化城乡规划的龙头作用，完成修编《城市总体规划纲要》、《新城区总体规划》、《城市综合交通体系规划》，完成主城区铁南片区、建国片区控制性详规，编制城市生态绿地系统、供热、停车场等专项规划，坚决维护规划的科学性和严肃性。

加大主城区建设力度。按照“三个一百万”人口布局，以“三城”同创为契机，加快建设区域中心城市。统筹实施城市建设、产业发展及民生改善行动计划，重点抓好主城区东西出口、北外环一级路、民主路立交桥等10大市政基础设施工程，启动建设城市展览馆、科技馆、图书馆等10大公建项目和12个特色博物馆，彰显科尔沁文化独特魅力。加大生态宜居和绿化力度，改造建设20个城市公园、30个社区公园和10条绿化带，改善人居环境。

提高城镇管理水平。开展10大市容环境整治专项行动，完善城镇老旧管网等市政基础设施，为市民营造文明整洁有序的生活环境。优先发展公共交通，改善城市交通环境，方便市民出行。支持社会资本参与城市基础设施和公共服务项目建设。加快建设智能城市管理数字化平台，推行精细化管理，培养引进城市管理人才，提高城市管理科学化、专业化水平。

以大开放促进县域经济大发展。加大对外开放力度，发挥市场跨地域、大范围配置资源、吸纳要素的决定性作用，充分利用国际国内两个市场、两种资源。跳出自身和自然条件限制，加快县域经济发展，增强县域经济实力。构建公路、铁路、民航立体交通网络，打造互联互通的开放大通道，以全方位、多层次、宽领域的对外开放格局，为县域经济的发展提供广阔空间。抓好霍林河综合资源循环经济和科尔沁自主创新与承接产业转移两大区域经济增长极，强化基础设施建设，通过开放聚集各类要素和项目，以贯通南北的经济走廊为骨干，形成北出南下、通关达海、融入东北经济一体化发展的县域经济开放新格局。北部加快发展铝新材料、现代煤化工产业;中部加快发展生物科技产业、文化旅游、现代金融和商贸物流等高端产业;南部“四点一带”加快发展绿色产业、精品旅游和不锈钢等非资源产业，打造各展其优、竞相发展的三大产业集群新高地。

(六)加强生态环境保护，推进生态文明建设

扎实抓好生态文明建设。祖国北方重要的生态安全屏障建设是我们义不容辞的责任。严格落实土地利用规划，实施总面积近7000万亩的五大生态功能区布局。按照主体功能区划定生态保护红线，明确生产、生活、生态空间开发管制界限，建立生态文明建设目标考核体系，加大对生态环境保护的考核力度。坚持源头治理、过程严管、后果严惩，完善环境治理和生态修复制度，为子孙后代留下蓝天绿地、碧水青山。

加大重点生态工程建设力度。加快科尔沁沙地综合治理等重点生态工程建设，完成科尔沁沙地治理45万亩，高标准新建城郊生态林24万亩，主城区近郊集中连片森林面积达到100万亩，完成道路绿化400公里。实施罕山封禁保护150万亩、草原休牧轮牧400万亩。加强生态自我修复和人工保护，修复生态植被48万亩，新建湿地自然保护小区39处。启动孟家段湿地和乌旦塔拉自然保护区的晋级申报工作，积极创建国家森林城市。落实长效机制，扎实开展“滥开垦、滥放牧、滥樵采”专项治理行动，确保生态管护措施真正落实到位。加大陡坡耕地、严重沙化耕地退耕退牧力度，还林还草140万亩。

注重资源节约和环境保护。以生态节水、生态草业、生态项目为重点，推进生态安全和环境友好型农牧业发展。实行最严格的水资源管理制度，突出抓好农业节水，压减农用机电井8300眼，农业节水4亿立方米以上，建设全国节水农业示范区。加大地下水污染防治力度，实施企业节水示范工程，实行企业用水效率考评和排放物严格达标管理制度。加强水源地保护和供水管网建设，提升供水能力和质量。推进居民生活用水阶梯水价制度，加快构建节水型社会。认真执行环保“三同时”制度，严把准入关，督促重点企业落实节能环保措施。加快矿山地质环境整治，建设和谐矿区。实施垃圾无害化处理，抓好城市污水处理、中水回用工程建设、运营和管理。强化大气污染防治，认真解决重金属、粉尘、白色污染等损害群众健康的突出问题，完成节能减排约束性指标。

(七)实施创新驱动，强化科技和人才支撑

推动科技与产业深度融合。充分发挥企业在科技创新中的主体作用，启动实施1000家科技小企业培育工程。支持铝新材料、玉米生物科技、现代蒙药、沙产业等优势产业组建创新联盟，推进科技成果产业化。抓好生物高科技创新园、科技大市场、蒙医药工程技术研究院、铝后新材料工程技术研究院四大科技平台建设。重视物联网、大数据等新科技的实际运用。进一步加强与清华大学、北京化工大学等高校和科研院所的合作，依靠科技创新推动经济转型升级。

实施人才强市工程。把人才工作摆在优先发展的战略位置，促进人口资源大市向人力资源强市转变，培养和造就一支与全市经济社会发展相适应的人才队伍。围绕县域经济、现代服务业、非物质文化传承、城市建设管理等方面，培养一批急需的实用人才。依托内蒙古民族大学、通辽职业学院、科尔沁职业艺术学院等院校，开设适应市场需求的专业课程，培养一批生产能手、经营能人和能工巧匠。新建一批博士后科研工作站，为实现经济社会可持续发展提供人才和智力支撑。

(八)创新文化体制机制，增强地区文化软实力

践行社会主义核心价值观。开展社会主义核心价值观教育活动，加强社会公德、职业道德、家庭美德和个人思想品德教育，推进社会诚信体系建设，扎实开展“学先进、树新风，做文明通辽人”等群众性精神文明和爱国卫生活动，提高公民道德素质和社会文明水平，积极创建全国文明城市和国家级卫生城市。

完善公共文化服务体系。围绕群众文化需求抓好文化惠民项目，建设基层综合性文化服务中心，引导和满足广大群众健康向上的精神文化需求。鼓励社会资本参与公共文化服务体系建设，推动公共文化服务社会化发展。加大非物质文化遗产和古遗址保护开发力度，大力传承和弘扬民族文化，加强对外文化宣传和交流，不断提升科尔沁文化的影响力。

加快文化产业发展。认真落实文化产业扶持政策，推进文化产业园和重点工程建设。深入挖掘特色文化资源，促进文化与旅游、金融、科技的融合发展，拓展延伸文化产业链条。鼓励民间资本与文化资源相结合，培育壮大文化龙头企业，推动文化产业发展。加强体育场馆建设，大力开展全民健身活动，积极备战自治区第十三届运动会。

(九)着力保障和改善民生，提高人民群众生活水平

做好扶贫开发工作。更加重视以产业脱贫、金融扶贫、生态移民为重点，打好扶贫攻坚战。启动金融扶贫富民工程，争取金融扶贫资金5.5亿元，按比例放大信贷规模。建设“四点一带”绿色经济产业带，发展优势特色产业，加快产业脱贫。加大整村推进、连片开发扶贫力度，扶贫规划、项目资金、帮扶干部到村到户，做到精准化扶贫。开展扶贫开发“回头看”，巩固扶贫成果。今年确保1.8万户、7万人实现稳定脱贫。

全面提升教育质量。统筹城乡义务教育资源，促进义务教育均衡发展。在农村牧区率先实现从幼儿园到高中的15年免费教育，健全经济困难学生资助体系，绝不能让贫困家庭学生因贫困辍学。优化教育布局，加大投入，加强基础设施建设，整合主城区教育资源，建立义务教育学校联盟，实行集团化管理。优先发展民族教育，完善民族教育体系，保持民族教育的领先地位。开工建设通辽蒙古族中学新校区。完善现代职业教育体系，推进主城区四校合并工作，培养符合产业需求和市场导向的实用人才。加强与北京西城区、东北三省优质教育资源的全面合作。实施名校长和名教师带动工程，加强教师和校长队伍建设，不断提高教育教学质量。

推进创业就业和社会保障工作。加快创业就业和社会保障信息平台建设，创建家庭服务业创业孵化基地、大学生创业园等4个创业就业基地，建设500个零散型创业孵化网点。新增城镇就业1.6万人、农牧民转移就业46万人。发放创业小额担保贷款2.2亿元。政府购买基层公共管理和社会服务岗位4000个，促进高校毕业生、城镇困难人员和退役军人就业。做好企业退休人员基本养老保险标准调整工作。完善社会救助制度，稳步提高城乡低保、农村牧区“五保”和城乡医疗救助水平。扩大基本医疗保险覆盖面，完善新农合制度，提高补助标准和核销比例，扩大重大疾病保障范围，努力让低收入百姓生活得更加舒心、更有尊严。

扎实推进百姓安居工程。加快保障性住房建设和棚户区改造，着力解决城镇中低收入家庭住房困难问题。新建各类保障性安居工程3.37万户，其中棚户区、城中村、城边村征收2.8万户。改造农村牧区危房2.5万户。

提高医疗卫生和健康服务水平。支持市医院做大做强，推进旗县医院达标晋级;加强与北京医管局和北京同仁医院、朝阳医院等医疗机构的帮扶合作，加快建立医疗联盟;完善基层医疗卫生服务体系，新建30所社区卫生服务站，实现社区医疗全覆盖，新改扩建10所贫困地区卫生院，对乡村医生免费培训;强化蒙中医机构服务能力和自治区级名院名科建设，实施蒙药制剂标准化，建设全国蒙医药发展示范区。广泛开展全民健康活动，深入进行城乡卫生环境专项治理，提高群众健康水平。

加强和创新社会治理。创新社会治安防控体系，依法防范和惩治违法犯罪活动。完善和创新流动人口、农村牧区“三留守”人员、特殊人群管理服务。全面实行重大决策社会稳定风险评估制度，有效化解社会矛盾和纠纷。全面落实安全生产责任制，深入开展“打非治违”专项行动，安全生产形势稳定好转。建立最严格的食品药品监管制度。做好气象、地震、人防等方面的应急管理工作，提高防灾减灾和应对突发事件能力。推进法治通辽建设，深化普法依法治理工作。支持军队建设，抓好国防动员和国防后备力量建设。深入开展民族团结进步宣传教育活动，精心做好民族工作，争创全国民族团结进步示范市。

(十)加快转变政府职能，创新行政管理服务方式

认真开展第二批党的群众路线教育实践活动。与推进改革发展工作相结合，推动树立形象、提振精神和凝心聚力相结合，增强宗旨意识、进取意识、机遇意识、责任意识，以更加务实的作风和明显的工作成效，汇聚起推动改革发展的正能量。

加快转变政府职能。理顺政府与市场的关系，进一步简政放权，公开市政府各部门行政审批事项清单，实行“阳光审批”。对市场机制能够有效调节的经济活动，一律取消审批;对列入自治区“8337”发展思路规划的重点项目，除必须经国家审批的，一律改为备案制;没有专门审批层次要求，由旗县管理更方便有效的事项，一律下放到旗县管理;对凡是企业投资的项目，除国家和自治区有明确规定外，一律由企业依法依规自主决策。对保留的行政审批事项，编制目录向社会公开，提高办事透明度。

创新政府管理和服务方式。加强发展战略、规划、政策、标准的制定和实施，更加注重对市场的监管和各类公共服务的提供。构建发展成果考核评价体系，关注劳动就业、居民收入、社会保障、人民健康状况，提升政府公共管理和服务水平。

提高依法行政水平。规范行政决策程序，推行重大行政决策听证制度。深化行政执法体制改革，完善执法程序，制定行政处罚裁量基准制度。自觉接受人大及其常委会、人民政协和社会各界的监督，广泛听取民主党派、工商联和无党派人士的意见。

强化对行政权力的制约监督。坚持用制度管权管事管人，严格执行“三重一大”制度，把权力关进制度的笼子。加强行政监察和审计监督，深入开展执法监察、效能监察和廉政监察。加强对领导干部经济决策、经济管理等经济责任方面的审计监督。

深入推进廉政建设和反腐败工作。严格执行中央“八项规定”和《党政机关厉行节约反对浪费条例》等一系列规定要求，坚决反对和纠正“四风”，进一步压减“三公”经费和一般性支出。加强地方政府性债务管理，坚持量入为出，量力而行。落实“一岗双责”，健全惩治和预防腐败体系，建设为民务实清廉百姓满意的政府。

各位代表，党的十八届三中全会开启了全面深化改革和现代化建设的新征程。担当更关键，行动最有力。让我们在市委的正确领导下，团结和带领全市各族干部群众，改革创新，锐意进取，用钉钉子的精神抓好各项工作落实，为开创通辽转型发展、科学发展、率先发展的新局面而努力奋斗!
